# Improved consistency of bond-line thickness when conducting single lap-shear joint tests

**DOI:** 10.1016/j.mex.2019.09.002

**Published:** 2019-09-10

**Authors:** Joe Flanagan, Lorcan Byrne, Joseph Mohan, Barry Twomey, Kenneth T. Stanton

**Affiliations:** a School of Mechanical & Materials Engineering, University College Dublin, Belfield, Dublin 4, Ireland; b ENBIO Ltd., DCU Alpha Innovation Campus, Glasnevin, Dublin 11, Ireland

**Keywords:** A novel method using a specially designed & coated bond-rig to improve the consistency and accuracy of sample lay-up when bonding samples for lap-shear testing, Adhesion, Lap-shear, Tensile testing, Release coating, Automotive, Aerospace

## Abstract

This article details a method for improving the consistency of bond-line thickness during lap-shear sample preparation. This includes the schematic for a lap-shear sample test rig and consideration for controlled variation of the bond-line thickness for up to ten pairs of samples at a time. Concerns regarding the curing of the samples when held on a large heat reservoir are addressed through direct measurement of the bond-rig temperature in combination with the cure chamber temperature. Additionally, the application of a release coating to the bond-rig has been demonstrated to improve ease of sample removal for the bond-rig, minimizing potential damage to the lap-shear sample set before testing. The release coating provides a clean surface for subsequent sets of samples, ensuring an even surface and reducing cleaning and degradation of the machined geometries of the rig. Overall, the proposed bond-rig provides:

• Increased bond-line uniformity
• Up to ten samples prepared in a batch
• Option to apply a release coating to improve usability and minimize cleaning

## Graphical abstract

**Figure:**
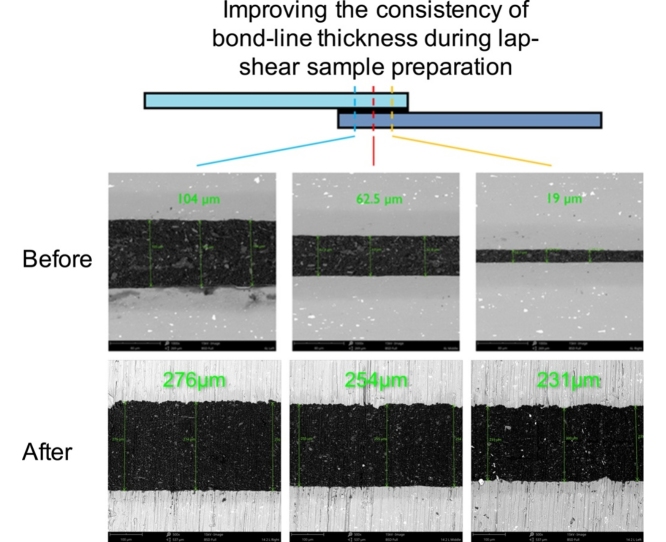

**Specifications Table**

**Table:** 

Subject Area:	Material Science
More specific subject area:	Adhesion Testing
Method name:	A novel method using a specially designed & coated bond-rig to improve the consistency and accuracy of sample lay-up when bonding samples for lap-shear testing.
Name and reference of original method:	ASTM D1002-10: Standard Test Method for Apparent Shear Strength of Single-Lap-Joint Adhesively Bonded Metal Specimens by Tension Loading (Metal-to- Metal)
Resource availability:	Drawings and. stp files for the bond-rig:
	DOI: https://doi.org/10.5281/zenodo.2530742 – ‘Lap-shear testing bond-rig design’

## Method details

Despite its recent criticism, single lap-shear joint testing (SLJ) is still the most commonly used method for the characterization of adhesive joint behavior and strength in industries such as the automotive, aerospace and oil & gas [1]. The SLJ test configuration is often criticized due to the number of setup parameters that can negatively affect the consistency of the test results unless controlled. Two such key parameters are correct alignment of the substrates and controlling the bond-line thickness of the adhesive. Due to the fact that the shear and peel stress distributions are concentrated at the edge of the joint overlap area, ensuring the joint maintains its rectangular shape with no overfill or under-fill at these free edges is imperative to maintain consistency with this joint setup [2]. This method aims to ensure correct alignment and consistent bond-line thickness are maintained with every series of tests, therefore reducing the likelihood of error for single batches and for batch-to-batch comparisons.

**Fig. 1:**
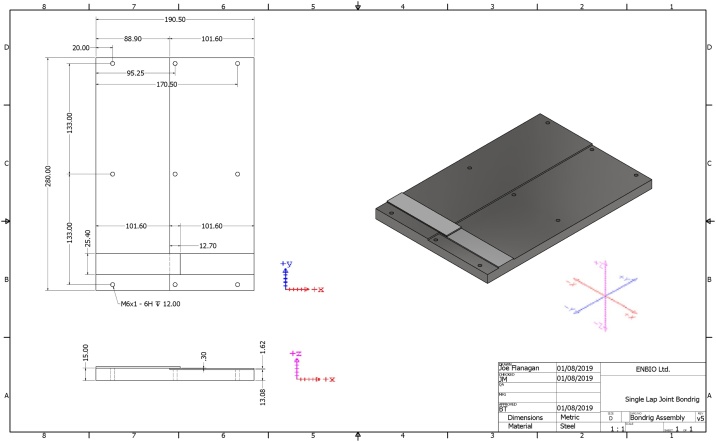
CAD model of the bond-rig design to ensure correct sample alignment and bond-line thickness.

Similar to the recommendations in the original standard [3], the method involved preparing the test joints in panels of at least five bonded samples. For this method, a bond-rig was designed which was able to accommodate ten bonded lap shear samples per test (the. stp files are included in the Resource material). This new bond-rig design ensured correct alignment of the samples and controlled the bond-line thickness of each sample. The design also ensured that the fillet at the edge of the adhesive joint are the same dimensions from batch-to-batch therefore ensuring that the geometric parameters that may influence bond strength of each bonded sample are kept consistent. `As can be seen from the CAD model of the new bond-rig in Fig. 1, the bond overlap length was controlled at 12.7 mm as per ASTM D1002-10 [3]. Each lap shear coupon was 101.6 mm in length, therefore by aligning each sample with the +*x* and –*x* edges of the bond-rig the overlap length was controlled at 12.7 mm. As can be seen in Fig. 1, the top level was 1.92 mm higher than the bottom level. This gave a bond-line thickness of 0.3 mm when taking into account the thickness of the lap shear samples (1.62 mm). This bond-line thickness was selected as it fell into the optimal bond-line thickness range of the 2-part Scotchweld 2216 epoxy adhesive used in the early parts of this work, as recommended by the manufacturer, 3 M. It was also found to be suitable for a 1-part epoxy film adhesive used later, namely Hexcel 300 g/m^2^ Redux 312.

With the bond-rig designed to control the bond-line thickness and sample alignment a number of samples were bonded to determine if the new design had improved the consistency of the joints. Previous bonding studies utilized a 15 μm non-stick release film to prevent any samples sticking to the bond-rig as a result of adhesive over-fill once pressure was applied. However, wrinkling of the non-stick release film during curing was found (see Supplementary material) to have a negative effect on maintaining a consistent bond-line thickness in the joint and this was evident after several curing cycles. To improve consistency and remove this effect, a CoBlast FEP (Fluorinated Ethylene Propylene) release coating was deposited on the parts. The process of depositing a fluoropolymer using CoBlast has previously been reported for the deposition of lubricious coatings on superelastic nitinol for medical device applications [4]. The application of this release layer eliminates the need for any release films or sprays. This one-step surface treatment greatly improves the accuracy of the bond-line thickness and is an optional improvement on the recommended joint setup in the original method [3].

**Fig. 2:**
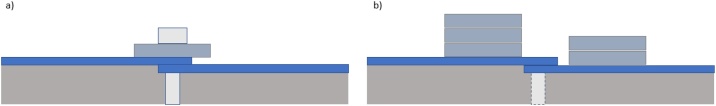
(a) Initial clamping setup using the alignment holes and (b) weighted clamping setup presented along x-axis.

The method of clamping the bonded samples was investigated as there were inconsistencies in the bond-line thickness depending on the position of the samples in the bond-rig. Two clamping methods were investigated as can be seen in Fig. 2(a) and (b).

**Fig. 3:**
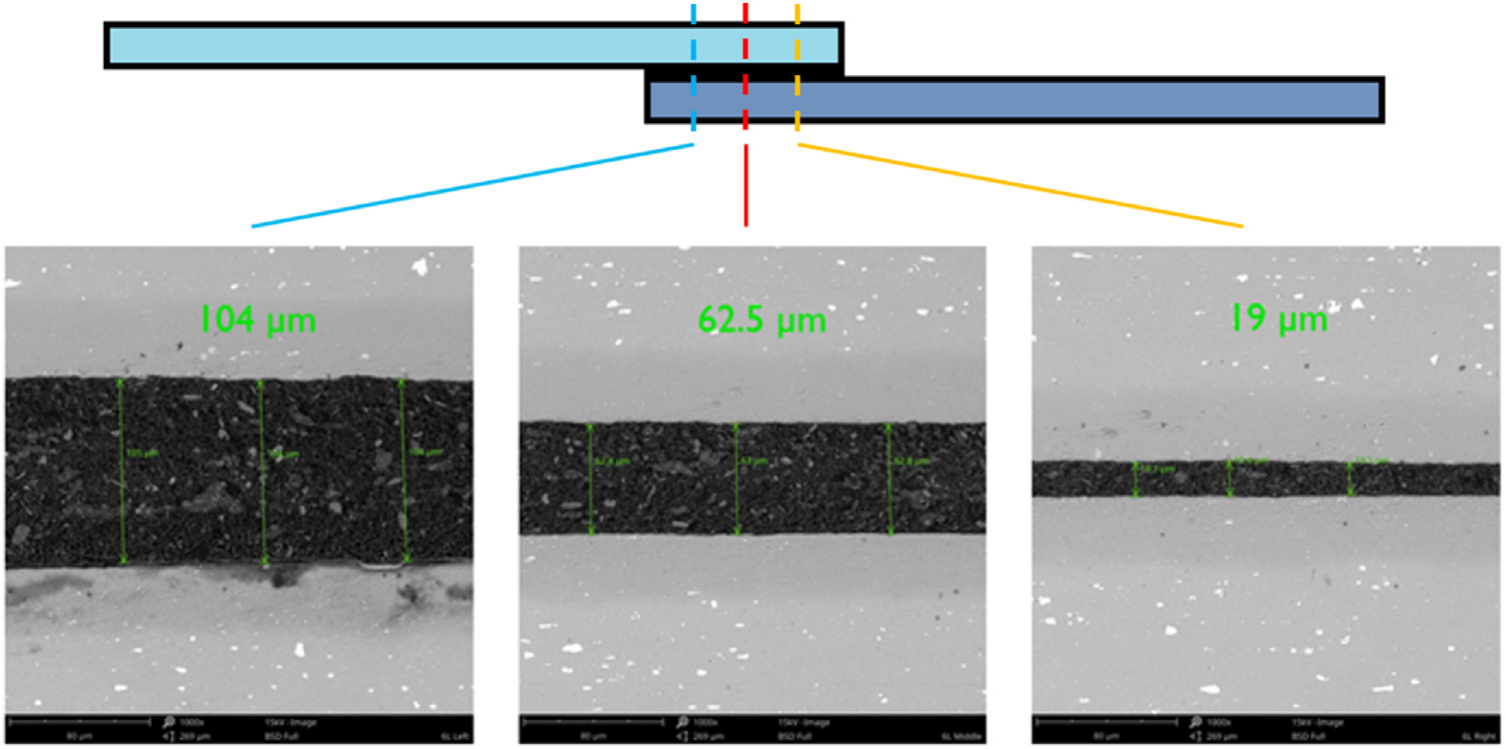
SEM cross sections of the ‘average’ bond-line for lap-shear joints bonded using the clamping method (a) imaged along x-axis.

The initial setup (Fig. 2(a)) utilized drilled and tapped holes as an anchor-point to secure a clamping bar which ran the full length of the bond area. However, it was found this resulted in an uneven bond-line within any given sample, with thickness increasing from left to right, as per Fig. 3. This was attributed to the unsupported overhang of the clamping arm on the right hand side causing additional squeeze-out, as compared to the supported left side (± 34% variation from average value). It was also noted that hand-tightening of the clamping arm screws resulted in varying bond-line thicknesses, often below the anticipated 300 μm.

In order to obtain a more consistent bond-line thickness both within and between samples, an alternative clamping arrangement was investigated, as per Fig. 2(b). Here, weights are placed along the bonding rig running the full length of the y-direction. These weights exerted a uniform force on each bonded sample during the cure cycle. This approach was found to significantly reduce the variation of bond-line thickness between samples, with an average bond-line thickness of 254 μm falling within the desired range. The variation of bond-line thickness within any given sample was reduced to less than 10% of the average thickness. This second approach was successfully implemented in a recent study comparing surface preparation techniques and finishes of bond-joints [5] (Supplementary Fig. S1).

## Conclusion

By ensuring equal weight distribution and using a bond-rig as detailed in Fig. 1 it is possible to greatly improve the consistency of the setup parameters and reduce the likelihood of over or under-fill in the joint. The additional use of the CoBlast FEP release coating further improves the setup process, ensuring that the joint maintains its rectangular shape along with a consistent bond-line thickness across the test panels. The release surface has been used extensively over the course of a year without issue (over 100 cure cycles); the samples can still be removed with ease with very little wear or adhesion to the surface evident. Noticeable improvements have also been observed in the consistency of bond strength results, demonstrating that once the setup parameters are maintained consistent, the single lap-shear joint test can provide very valuable information on the behavior and strength of adhesive joints and reduce user effects. Furthermore, the presence of bond-line defects such as porosity or micro-voids could be eliminated by incorporating a vacuum bagging system with the bond-rig discussed here within. This was not included in the present work due to time constraints but is recommended for future work if more accuracy is required.
