# Supplementary material for: Improved consistency of bond-line thickness when conducting single lap-shear joint tests
Source: MethodsX. 2019 Sep 10;6:1974–8. doi: 10.1016/j.mex.2019.09.002 (PMC6854070; doi:10.1016/j.mex.2019.09.002)
Supplement: Supplementary file 1 [file mmc1.docx]

**Supplementary material *and/or* Additional information:**

As per clamping setup (a) in Fig. 2, 15 μm non-stick release film was placed on the bond-rig to prevent any samples sticking to the bond-rig and clamping bars (Fig. S1). On the bond-rig plate side of the joint, wrinkling of the non-stick release film was observed and found to impact on the consistency of the bonded joints. This reduced consistency within batches and from batch-to-batch.


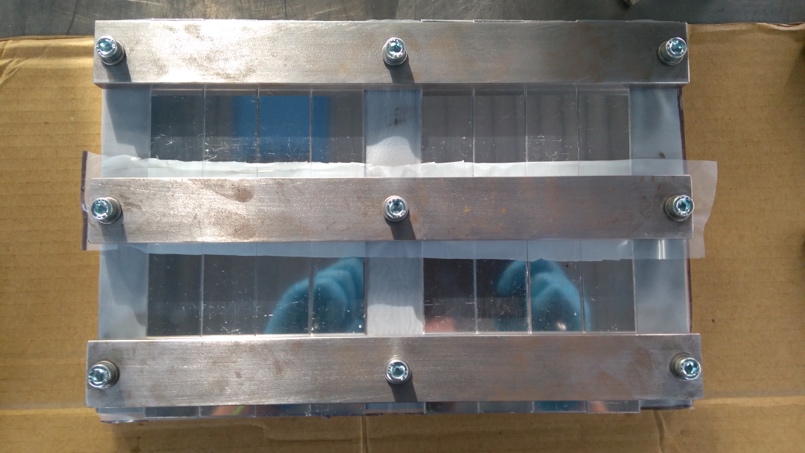

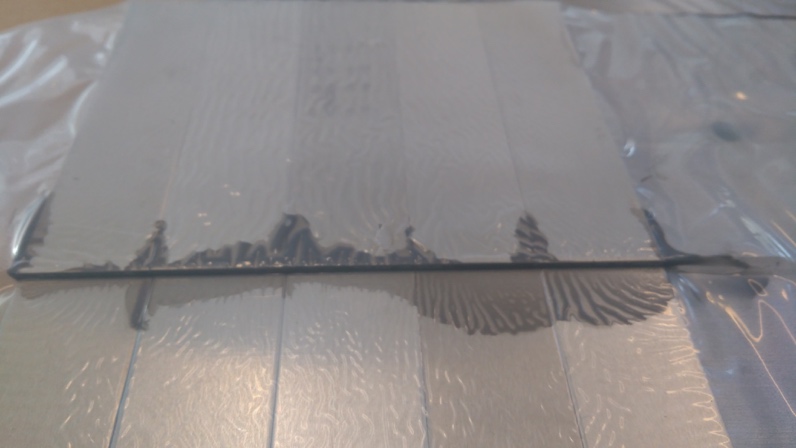


Figure S1: Image of the bond-rig with clamps secured in place (a) and the bonded samples with excessive overflow sticking to the inside of the release film (b). (x-axis top-to-bottom; y-axis left to right)
